# Topics most predictive of favorable overall assessment in outpatient radiology

**DOI:** 10.1371/journal.pone.0285288

**Published:** 2023-05-03

**Authors:** Amna A. Ajam, Colin Berkheimer, Bin Xing, Aadil Umerani, Shayaan Rasheed, Xuan V. Nguyen

**Affiliations:** 1 Department of Radiology, The Ohio State University College of Medicine, Columbus, OH, United States of America; 2 Lake Erie College of Osteopathic Medicine, Erie, PA, United States of America; 3 GE Healthcare, Waukesha, WI, United States of America; 4 The Ohio State University, Columbus, OH, United States of America; Hosei University: Hosei Daigaku, JAPAN

## Abstract

**Background:**

Patients’ subjective experiences during clinical interactions may affect their engagement in healthcare, and better understanding of the issues patients consider most important may help improve service quality and patient-staff relationships. While diagnostic imaging is a growing component of healthcare utilization, few studies have quantitatively and systematically assessed what patients deem most relevant in radiology settings. To elucidate factors driving patient satisfaction in outpatient radiology, we derived quantitative models to identify items most predictive of patients’ overall assessment of radiology encounters.

**Methods:**

Press-Ganey survey data (N = 69,319) collected over a 9-year period at a single institution were retrospectively analyzed, with each item response dichotomized as “favorable” or “unfavorable.” Multiple logistic regression analyses were performed on 18 binarized Likert items to compute odds ratios (OR) for those question items significantly predicting Overall Rating of Care or Likelihood of Recommending. In a secondary analysis to identify topics more relevant to radiology than other encounter types, items significantly more predictive of concordant ratings in radiology compared to non-radiology visits were also identified.

**Results:**

Among radiology survey respondents, top predictors of Overall Rating and Likelihood of Recommending were items addressing patient concerns or complaints (OR 6.8 and 4.9, respectively) and sensitivity to patient needs (OR 4.7 and 4.5, respectively). When comparing radiology and non-radiology visits, the top items more predictive for radiology included unfavorable responses to helpfulness of registration desk personnel (OR 1.4–1.6), comfort of waiting areas (OR 1.4), and ease of obtaining an appointment at the desired time (OR 1.4).

**Conclusions:**

Items related to patient-centered empathic communication were the most predictive of favorable overall ratings among radiology outpatients, while underperformance in logistical issues related to registration, scheduling, and waiting areas may have greater adverse impact on radiology than non-radiology encounters. Findings may offer potential targets for future quality improvement efforts.

## Introduction

Patient-centered care, in which healthcare providers identify and meet patients’ needs and preferences, is an increasingly important facet of healthcare. With recent implementation of quality payment programs such as the Merit-Based Incentive Payment System (MIPS), radiology practices and other healthcare entities are increasingly incentivized to demonstrate increased value or better outcomes [1–3], including patient satisfaction and metrics related to patient-centered care [1]. Additionally, patient satisfaction may contribute to the attraction and/or retention of patients in radiology [4] and other healthcare settings [5–9]. Given the variety of options available to improve the patient experience in radiology, particularly in challenging situations in MRI or interventional procedures [10–14], an understanding of which components of a patient experience are most crucial to patients’ overall satisfaction or willingness to recommend is an important goal in clinical radiology practice.

Existing studies attempting to elucidate the dominant contributors to radiology patient satisfaction based on specific quality improvement initiatives, analyses of complaints and targeted feedback, or post-encounter surveys revealed several themes found important to patients. An early qualitative study of written complaints in radiology determined that failure to provide patient-centered care and poor interpersonal interactions with staff were common themes among submitted complaints [15]. Standardized surveys, such as those developed by Press-Ganey (PG), have also been analyzed to assess various components of patient satisfaction following radiology quality improvement initiatives [16]. Surveys targeting patient communication found that patients are most concerned with receiving adequate information to prepare for their imaging studies [17]. Another study utilizing electric kiosks to survey patients following their visit at a tertiary-care imaging center revealed that wait times, cleanliness, receptionist courtesy, and patient-staff communication were most important [18]. A published observation that short medical history interviews improved patient satisfaction in radiology practice settings implied that patient-centered communication is a major contributor of radiology patient satisfaction [19]. A 2019 European Society of Radiology survey found that patients valued correctness of diagnosis and communication from medical staff the most [20]. Many of the above studies were limited by relatively small sample sizes or a relatively short duration of sampling.

While many existing studies simply report satisfaction scores for various domains or subtopics akin to a quality assessment report, our approach differs in that we seek to quantify the relative importance of each question item on the target outcome variables of interest using a multivariable statistical prediction model. Insights gleaned from a systematic, data-driven approach to identifying top predictors of patient satisfaction outcomes may facilitate prioritization of practice improvements deemed of highest potential yield for a given practice and patient population. In this study, we derive two quantitative models for predicting the two dependent variables of overall subjective ratings and willingness to recommend to others as a function of individual Press-Ganey survey question items. We further quantify the impact of specific topics within two population subgroups that had been observed in a prior publication to have a lower satisfaction with their radiology care (young working-age patients and certain racial minority groups) [21]. Moreover, we test in a secondary analysis the hypothesis that some items are more predictive of the outcomes of interest in radiology vs. non-radiology outpatient visits.

## Methods

### Survey data

In this IRB-approved and HIPAA-compliant study, PG surveys completed over a period of 9 years (2008–2017) at a single institution were retrospectively analyzed. IRB approval was obtained from the Biomedical Sciences Institutional Review Board of the Ohio State University (Columbus, OH), and informed consent was waived due to anonymized analysis of previously acquired data. The PG surveys consisted of multiple Likert items grouped into various sections that asked participants to rank various aspects of their experience following their healthcare visit, such as ease of scheduling an appointment, responsiveness of staff to patient complaints and sensitivity to their needs, skill of the staff, and wait times. Excluding the Overall Assessment section, there were 18 question items distributed among the following four sections: Registration, Facilities, Test or Treatment, and Personal Issues [4]. The items were answered using a 5-point rating scale, with 1 representing a “Very Poor” response and 5 representing a “Very Good” response. To dichotomize survey results, responses of 4–5 were classified as favorable, while responses of 1–3 were classified as unfavorable. Within the Overall Assessment section, 2 items were selected to serve as relevant outcome variables: the patient’s subjective overall rating of care (Overall) and the likelihood of recommending a center to others (Recommend). All surveys in the study period were included in the statistical analysis, including those that were partially completed.

### Statistics

Missing data in the survey were addressed with multiple imputation (MI) under the missing at random (MAR) assumption, which stipulates that the probabilities of data being missing are independent of the missing values and that the systematic differences between the missing and observed values can be entirely explained by other observed variables [22], including all outcome and predictor variables. The logistic regression analyses were performed separately for five MI instances of the dataset, and final statistical inferences were drawn via combining the results from each MI repetition. Since the data are not distributed normally, a binary logistic regression model was adopted to fit the model, with the binarized Recommend and Overall variables as dependent variables and 18 binarized questionnaire items as predictor variables. Specifically, the variables were selected with a stepwise approach in SAS base 9.4 (SAS Institute Inc., Cary, NC). The variable candidates enter the model based on evaluation of the significance level at 0.20 in each complete dataset. In the second step, all the selected variables were employed in a logistic regression model using the GENMOD procedure in SAS to estimate odds ratios (OR), 95% confidence intervals, and p-values for each predictor variable. Statistically significant results were determined based on p < 0.05, with Bonferroni adjustment for multiple comparisons.

We applied a similar regression model selection approach to identify predictor variables most predictive of favorable outcomes for demographic subsets previously observed to demonstrate significantly lower satisfaction rates following radiology appointments [21]. The two population groups examined were those aged 20–39 (Age) and those of Asian, black, or other/unknown race (Race). As before, eighteen independent predictor variables and one outcome variable were included for initial variable selection, followed by multiple logistic regression analysis performed to estimate odds ratios, 95% confidence intervals, and p-values for each subgroup.

With the same two-step approach, SAS procedure, and method, the hypothesis that the odds ratios of the positive responses for the Recommend and Overall variables between radiology and non-radiology populations at each level of the binary predictor variables are different from 1 was tested in a logistic regression analysis using the GENMOD procedure, with a SLICE statement for data partitioning. Specifically, the 18 predictor variables and one outcome variable were included for initial selection in the model, followed by multiple logistic regression analysis using the group variable (i.e., radiology or non-radiology), selected individual variables, and interaction terms as predictors in the model. The odds ratio of the Recommend and Overall variables for radiology vs. non-radiology populations at each level of the binary predictor variables was calculated using each imputed dataset, followed by the MIANALYZE procedure to compute summarized odds ratios. From this, the concordance between outcome and predictor variables (i.e., both favorable or both unfavorable) was able to be compared between radiology and non-radiology patients. The 95% confidence intervals and p-values were computed with Bonferroni adjustment for multiple comparisons.

## Results

A total of 69,319 survey were analyzed, of which 36,693 were completed by patients following a radiology appointment and 32,626 were completed by patients following a non-radiology appointment. Of the non-radiology surveys, 763 (2.4%) contained unfavorable responses to overall rating of care and 1,049 (3.3%) had unfavorable responses to likelihood of recommending a center to others (Table 1). Of the radiology surveys, 887 (2.4%) had unfavorable responses to overall rating and 1,136 (3.1%) showed unfavorable responses to likelihood of recommending (Table 1).

**Table 1:** Number of surveys having either a favorable rating or unfavorable rating amongst radiology and non-radiology visits.

		Overall Rating			Recommend	
	Favorable	Unfavorable	% unfavorable	Favorable	Unfavorable	% unfavorable
Non-Radiology	31446	763	2.4%	31053	1049	3.3%
Radiology	35330	887	2.4%	34955	1136	3.1%

Table 2 lists the counts and percentages of missing responses for radiology and non-radiology visits. The question items are shown in Table 2 as worded in the survey and in the order they appear in the survey. The entries with the highest missing percentages include items related to keeping family informed, responsiveness to concerns/complaints, test/treatment preparation instructions, waiting time in the test/treatment area, and ease of obtaining an appointment. All these question items with high missing frequencies can plausibly be explained by encounters for which these items are “not applicable,” such as when no family is present, no specific concerns/complaints exist, no specific preparation is needed, no separate waiting in test/treatment areas occurs, or care is provided on a walk-in basis, respectively. While missing responses occur more frequently with some questions than others or with differences in frequency between radiology and non-radiology visits, our missing at random assumption likely remains valid because there are unlikely to be any systematic differences between missing and observed values, as unanswered question items are mostly likely due to the patient being indifferent in their assessment or deeming the issue to be irrelevant or unimportant.

**Table 2:** Frequency of missing responses for survey question items.

	Non-radiology		Radiology	
Total	32,626		36,693	
	Missing count	Missing %	Missing count	Missing %
Ease of getting an appointment when you wanted	20,276	62.1%	2,531	6.9%
Helpfulness of the person at the registration desk	463	1.4%	404	1.1%
Ease of the registration process	561	1.7%	479	1.3%
Waiting time in registration	720	2.2%	694	1.9%
Comfort of the waiting area	335	1.0%	278	0.8%
Ease of finding your way around	481	1.5%	392	1.1%
Cleanliness of facility	491	1.5%	416	1.1%
Instructions you received to prepare for your test or treatment (if any)	21,032	64.5%	4,191	11.4%
Time you spent waiting in this test or treatment area	20,369	62.4%	2,421	6.6%
Friendliness/courtesy of the staff who provided your test or treatment	453	1.4%	472	1.3%
Explanations from the staff about what would happen during your test or treatment	668	2.0%	671	1.8%
Skill of the staff who provided your test or treatment	590	1.8%	593	1.6%
Staff concern for your comfort	563	1.7%	580	1.6%
Staff’s concern for your questions and worries	784	2.4%	1,422	3.9%
Our concern for your privacy	768	2.4%	831	2.3%
Our sensitivity to your needs	719	2.2%	1,023	2.8%
Response to concerns/complaints made during your visit	2,930	9.0%	5,313	14.5%
Staff concern to keep your family informed about your test or treatment	21,458	65.8%	9,201	25.1%
Overall rating of care received during your visit	417	1.3%	476	1.3%
Likelihood of your recommending our facility to others	524	1.6%	602	1.6%

The logistic regression analysis applied to radiology appointments identified 9 survey items that independently predicted the overall rating of care and 12 survey items that independently predicted likelihood of recommending (Table 3). The strongest independent predictor for both outcome variables was responsiveness to concerns and complaints made during a visit (OR = 6.79, p = <0.0001 for Overall; OR = 4.94, p <0.0001 for Recommend). The next strongest predictor for both outcomes was sensitivity to the patient’s needs (OR = 4.66, p <0.0001] for Overall; OR = 4.46, p <0.0001 for Recommend). Skill level of the staff who provided care to the patient was also a predictor for both outcomes (OR = 4.33, p <0.0001 for Overall; OR = 2.73, p = 0.0005 for Recommend). Other question items that were independent predictor variables for both outcomes addressed wait times in the testing/treatment area, staff communications with patient and family about the test or treatments, helpfulness of registration personnel, friendliness or courtesy of the staff, comfort of the waiting area, and staff explanations regarding the test or treatment (OR range: 1.72–3.58) (Table 3).

**Table 3:** Survey question items most predictive of favorable “Overall Rating of Care” and “Likelihood of Recommending” for radiology appointments.

	Overall			Recommend		
	Odds Ratio (95% CI)	p value	Rank	Odds Ratio (95% CI)	p value	Rank
Question Description						
Response to concerns/complaints made during your visit	6.79 (4.65–9.92)	< .0001	1	4.94 (3.63–6.72)	< .0001	1
Our sensitivity to your needs	4.66 (3.29–6.61)	< .0001	2	4.46 (3.21–6.20)	< .0001	2
Skill of the staff who provided your test or treatment	4.33 (2.92–6.42)	< .0001	3	2.73 (1.88–3.97)	< .0001	3
Time you spent waiting in this test or treatment area	3.58 (2.75–4.65)	< .0001	4	2.27 (1.8–2.87)	< .0001	5
Helpfulness of the person at the registration desk	2.61 (1.76–3.85)	< .0001	5	1.87 (1.25–2.79)	0.0307	11
Friendliness/courtesy of the staff who provided your test or treatment	2.35 (1.54–3.60)	0.0011	6	1.91 (1.29–2.82)	0.0163	10
Staff concern for your comfort	2.20 (1.55–3.13)	0.0001	7	-------------------	-----	----
Comfort of the waiting area	2.16 (1.58–2.93)	< .0001	8	2.48 (1.88–3.26)	< .0001	4
Ease of the registration process	-------------------	------	----	2.15 (1.46–3.17)	0.0014	6
Cleanliness of facility	-------------------	------	----	2.07 (1.40–3.05)	0.0032	8
Ease of getting an appointment when you wanted	-------------------	------	----	2.07 (1.56–2.74)	< .0001	7
Ease of finding your way around	-------------------	------	----	1.96 (1.52–2.54)	< .0001	9
Explanations from the staff about what would happen during your test or treatment	1.76 (1.22–2.52)	0.0228	9	1.72 (1.21–2.44)	0.0303	12

Likelihood to recommend, but not overall rating, was independently predicted by favorable responses to items regarding cleanliness of the facility (OR = 2.07, p = 0.0032), ease of getting an appointment at the desired time (OR = 2.07, p <0.0001), ease of the registration process (OR = 2.15, p = 0.0014), and ease of navigating the facility (OR = 1.96, p <0.0001). Staff concern for patient comfort was a predictor of overall rating but not likelihood of recommending (OR = 2.20, p < 0.0001).

When applied to subgroups of the population that were previously observed to have lower radiology visit satisfaction, the logistic regression analysis identified 5 survey items in the targeted age subgroup and 5 items in the targeted racial minority subgroup that independently predicted the overall rating of care (Table 4). Response to concerns and complaints was a predictor in both groups (OR = 4.88, p = 0.018 for Age; OR = 8.68, p <0.0001 for Race). Other survey items predicting the overall rating of care in both groups included sensitivity to patients’ needs (OR = 5.97, p = 0.0016 for Age; OR = 5.09, p = 0.0004 for Race), waiting time in the testing and treatment area (OR = 4.06, p <0.0017 for Age; OR = 4.49, p < 0.0001 for Race), and friendliness of staff (OR = 3.34, p = 0.019 for Age; OR = 4.43, p = 0.0062 for Race). Staff concern for comfort (OR = 3.71, p = 0.012) was predictive for overall rating in the targeted age subgroup but were not predictive in the targeted race subgroup. Skill of nonphysician healthcare staff (OR = 4.23, p = 0.0080 for Race) was predictive in the targeted race subgroup but not in the targeted age subgroup.

**Table 4:** Survey question items most predictive of favorable “Overall Rating of Care” and “Likelihood of Recommending” for radiology appointments among demographic subgroups previously reported to have higher dissatisfaction rates.

Patients aged 20–39 years: Predictors of Overall Rating of Care		
Question Description	Odds Ratio (95% CI)	p value
Our sensitivity to your needs	5.97 (2.29–15.6)	0.0016
Response to concerns/complaints made during your visit	4.88 (1.71–13.92)	0.018
Time you spent waiting in this testing or treatment area	4.06 (1.97–8.38)	0.0017
Staff concern for your comfort	3.71 (1.62–8.48)	0.012
Friendliness/courtesy of the staff who provided your test or treatment	3.34 (1.5–7.43)	0.019
**Patients aged 20–39 years: Predictors of Likelihood of Recommending**		
**Question Description**	**Odds Ratio (95% CI)**	**p value**
Our sensitivity to your needs	9.23 (3.95–21.56)	< .0001
Explanations from the staff about what would happen during your test or treatment	6.77 (3.49–13.13)	< .0001
Response to concerns/complaints made during your visit	5.23 (2.07–13.2)	0.0038
Time spent waiting in this testing and treatment area	4.10 (2.19–7.70)	< .0001
**Patients identifying as Asian, Black, or Other/unknown races: Predictors of Overall Rating of Care**		
**Question Description**	**Odds Ratio (95% CI)**	**p value**
Response to concerns/complaints made during your visit	8.68 (3.80–19.82)	< .0001
Our sensitivity to your needs	5.09 (2.29–11.29)	0.0004
Time you spent waiting in this testing and treatment area	4.49 (2.35–8.58)	< .0001
Friendliness/courtesy of the staff who provided your test or treatment	4.43 (1.84–10.65)	0.0062
Skill of the staff who provided your test or treatment	4.23 (1.77–10.11)	0.0080
**Patients identifying as Asian, Black, or Other/unknown races: Predictors of Likelihood of Recommending**		
**Question Description**	**Odds Ratio (95% CI)**	**p value**
Response to concerns/complaints made during your visit	10.77 (4.56–25.43)	< .0001
Explanations from the staff about what would happen during your test or treatment	6.67 (3.58–12.43)	< .0001
Our sensitivity to your needs	5.23 (2.36–11.59)	0.0003

The logistic regression analysis for the population subgroups identified 4 different survey items in the targeted age subgroup and 3 different items in the targeted race subgroup that independently predicted the likelihood of recommending (Table 4). Sensitivity to patient needs was the strongest predictor for the targeted age subgroup (OR = 9.23, p <0.0001). Response to concerns and complaints was the strongest predictor variable for the targeted race subgroup (OR = 10.77, p <0.0001) and was also a strong predictor for the targeted age subgroup (OR = 5.23, p = 0.0038). Other predictive variables for both groups included explanations given by staff (OR = 6.77, p < 0.0001 for Age; OR = 6.67, p = <0.0001 for Race). The only predictive item in the targeted age subgroup that was not predictive in the targeted race subgroup was waiting time in the testing and treatment area (OR = 4.1, p <0.0001). All predictive items in the targeted race subgroup were also predictive in the targeted age subgroup.

An additional logistic regression analysis was performed to assess whether some survey items are more likely to predict a concordant outcome response for radiology visits compared to non-radiology visits (Table 5). An unfavorable response to helpfulness of registration desk personnel was more predictive of a concordant negative response to both outcome variables among radiology patients compared to non-radiology patients (OR = 1.55, p = 0.0002 for Overall; OR = 1.42, p = 0.0087 for Recommend). An unfavorable assessment of waiting area comfort was more predictive of a concordant unfavorable response to overall rating among radiology patients compared to non-radiology patients (OR = 1.36, p = 0.0054). Finally, an unfavorable assessment of ease of scheduling appointment was more predictive of a concordant unfavorable response to recommend rating among radiology patients compared to non-radiology patients (OR = 1.38, p = 0.0037).

**Table 5:** Survey question items that are more predictive of concordant outcomes for radiology appointments than for non-radiology appointments^†^.

**Concordance with Overall Rating of Care**			
**Question Description**	**Concordant Result**	**Odds Ratio (95% CI)**	**p value** *
Helpfulness of the person at the registration desk	Unfavorable	1.55 (1.28–1.89)	0.0002
Comfort of the waiting area	Unfavorable	1.36 (1.15–1.60)	0.0054
**Concordance with Likelihood of Recommending**			
**Question Description**	**Concordant Result**	**Odds Ratio (95% CI)**	**p value** *
Helpfulness of the person at the registration desk	Unfavorable	1.42 (1.17–1.72)	0.0087
Ease of getting an appointment when you wanted	Unfavorable	1.38 (1.17–1.62)	0.0037

^†^Odds ratios greater than 1 denote that for the same outcome variable (e.g., “Overall Rating of Care” or “Likelihood of Recommending”), the corresponding question item for the radiology visits are more likely to be concordant with the outcome shown than for non-radiology visits.

*p values shown represent adjusted p values for multiple comparisons upon Bonferroni correction, with the significance level set at 0.05.

## Discussion

In this large analysis of over tens of thousands of completed satisfaction surveys over a near-decade of practice, we were able to quantify the extent to which specific question items predict a favorable overall rating of care or likelihood of recommending to others. For both these outcome variables, the two most predictive question items address the practice’s responsiveness to patient concerns and complaints and its sensitivity to patient needs, illustrating a crucial role of empathic communication in patient experiences. Responsiveness to patient concerns is even more predictive when analyzing population subgroups, such as racial minorities, that had previously been noted to have lower satisfaction metrics in outpatient radiology. Responses to some survey items had a greater apparent impact on predicting a concordant outcome among radiology patients compared to non-radiology patients. For instance, negative responses regarding the helpfulness of the registration desk, ease of getting an appointment, and the comfort of waiting areas were more predictive of unfavorable outcome responses for radiology visits than for non-radiology visits, suggesting these topics are weighed by patients more heavily for radiology encounters than for non-radiology encounters.

Historically, topics and issues most important to patients can be identified using qualitative approaches such as focus group sessions, targeted requests for feedback, or using more structured methods such as surveys and questionnaires. However, our approach to employ statistical modelling on a large database of existing survey responses to objectively assess odds ratios for individual question items represents an efficient method for assessing the major contributing issues affecting patient dissatisfaction or satisfaction. Prior investigations utilizing either electronic or paper surveys have reported that patient satisfaction in radiology is strongly influenced by factors related to patient-staff communication [17–20]. One analysis of patient complaints found a majority were related to failure of patient-centered care [15]. Studies using Press-Ganey satisfaction surveys in orthopedic surgery and orthopedic oncology have also found that interpersonal and communication issues and sensitivity to patient needs were influential factors predicting patient satisfaction and likelihood of recommending a practice [23, 24]. The results from our quantitative model, that the most influential predictors of favorable patient experience were responsiveness to patients’ concerns or complaints and sensitivity to patient’s needs, are concordant with previously published findings that highlight the importance of a patient-centered culture and a need for appropriate communication skills to optimize patient experiences.

Our results highlight the opportunity of existing satisfaction survey instruments to provide more than simply a quality outcome metric. By performing a data-driven analysis quantifying impact of contributors to patients’ overall assessment or likelihood of recommending a practice, issues that most impact their experience may be pinpointed. Consequently, items with high odds ratios predicting favorable experiences could be interpreted as high-yield targets for focused quality improvement initiatives. In our study, where the most predictive factors are related to patient-centered empathic communication, there have been reported benefits of staff communication training on patient satisfaction [1]. Quality improvement initiatives may also be selectively applied to specific subgroups of the patient population, allowing for more focused outreach and targeted interventions to improve satisfaction in subgroups of patients with historically suboptimal satisfaction scores. Furthermore, although determinants of patient satisfaction in radiology are likely to overlap with those for non-radiology encounters, the comparison between radiology and non-radiology survey responses with respect to prediction of concordant outcomes discussed in our study could help identify topics that may deserve more attention in radiology, such as logistical items related to registration, scheduling, and waiting areas as identified in our study.

This study has several limitations. As a single-institution study, there may be limited generalizability to other institutions, other practice settings, or other patient populations. Nonetheless, it is plausible that patient expectations and considerations in the healthcare setting and consequently the quantitative relationships examined in our study may be quite similar across institutions. Another potential limitation is that the methodological approach to identify independent predictors within the statistical model may not necessarily detect all relevant survey question items if some correlate with other independent predictors. As with any survey-based approach, there is potential for non-response bias among patient populations when utilizing PG survey instruments [25], such that the population responding to surveys in our study may not necessarily reflect the entire radiology patient population. While it is possible that such variables as illness severity could potentially affect a decision to respond to the surveys, the surveys were sent only to outpatients; therefore, the most serious or life-threatening encounters, which typically result in hospital admissions, intensive care unit stays, or emergency department visits, are not included in the surveyed encounters. We did not record other variables that may explain patients’ non-participation in the survey, and non-response bias cannot be completely eliminated with survey-based methods, but we anticipate that the impact of such bias on our study’s interpretation is minimal.

## Conclusions

In summary, our study applied logistic regression to identify PG survey items most predictive of favorable patient responses for overall rating of care and likelihood of recommending. Items related to patient-centered empathic communication were the most predictive of favorable ratings among radiology patients, offering potential high-yield targets for future quality improvement programs. The relative impact of some of these topics is even greater when focusing on specific population subgroups previously demonstrated to have lower satisfaction ratings in outpatient radiology. When contrasting radiology and non-radiology encounters, underperformance in logistical issues related to registration, scheduling, and waiting areas may have greater adverse impact on patient satisfaction for radiology visits than for non-radiology encounters. In a healthcare landscape that is becoming more focused on patient-centered care, identification of drivers of patient satisfaction could become a valuable tool in maximizing both practice reimbursement and patient retention. Further investigations into the drivers of patient satisfaction and the impact of focused quality improvement initiatives in specific radiology settings would be helpful for ongoing efforts to improve the radiology patient experience.

## Supporting information

S1 Dataset (XLSX)

## References

[1] Lang EV, Yuh WT, Ajam A, Kelly R, Macadam L, Potts R, et al. Understanding patient satisfaction ratings for radiology services. AJR Am J Roentgenol. 2013;201: 1190–1196. doi: 10.2214/AJR.13.11281 24261356 PMC4427188

[2] Golding LP, Nicola GN, Duszak R Jr, Rosenkrantz AB. Facility-Based Measurement in the Merit-Based Incentive Payment System: A Potential Safety Net for Which Most Radiologists Will Be Eligible. AJR Am J Roentgenol. 2019;213: 998–1002. doi: 10.2214/AJR.19.21344 31180736

[3] Rosenkrantz AB, Goldberg JE, Duszak R Jr, Nicola GN. Merit-Based Incentive Payment System Participation: Radiologists Can Run but Cannot Hide. J Am Coll Radiol. 2018;15: 674–680. doi: 10.1016/j.jacr.2017.11.009 29254885

[4] Ajam AA, Lang EV, Nguyen XV. Does Patient Satisfaction Drive Volumes in Outpatient Magnetic Resonance Imaging? Curr Probl Diagn Radiol. 2021. doi: 10.1067/j.cpradiol.2021.09.005 34887134 PMC9091048

[5] Sun BC, Adams J, Orav EJ, Rucker DW, Brennan TA, Burstin HR. Determinants of patient satisfaction and willingness to return with emergency care. Ann Emerg Med. 2000;35: 426–434. 10783404

[6] Schaal T, Schoenfelder T, Klewer J, Kugler J. Determinants of patient satisfaction and their willingness to return after primary total hip replacement: a cross-sectional study. BMC Musculoskelet Disord. 2016;17: 330-016–1196-3. doi: 10.1186/s12891-016-1196-3 27502761 PMC4977730

[7] Burroughs TE, Davies AR, Cira JC, Dunagan WC. Understanding patient willingness to recommend and return: a strategy for prioritizing improvement opportunities. Jt Comm J Qual Improv. 1999;25: 271–287. doi: 10.1016/s1070-3241(16)30444-8 10367265

[8] Otani K, Waterman B, Faulkner KM, Boslaugh S, Dunagan WC. How patient reactions to hospital care attributes affect the evaluation of overall quality of care, willingness to recommend, and willingness to return. J Healthc Manag. 2010;55: 25–37; discussion 38. 20210071

[9] Kim CE, Shin JS, Lee J, Lee YJ, Kim MR, Choi A, et al. Quality of medical service, patient satisfaction and loyalty with a focus on interpersonal-based medical service encounters and treatment effectiveness: a cross-sectional multicenter study of complementary and alternative medicine (CAM) hospitals. BMC Complement Altern Med. 2017;17: 174-017–1691-6. doi: 10.1186/s12906-017-1691-6 28351389 PMC5370429

[10] Nguyen XV, Tahir S, Bresnahan BW, Andre JB, Lang EV, Mossa-Basha M, et al. Prevalence and Financial Impact of Claustrophobia, Anxiety, Patient Motion, and Other Patient Events in Magnetic Resonance Imaging. Top Magn Reson Imaging. 2020;29: 125–130. doi: 10.1097/RMR.0000000000000243 32568974

[11] Brunnquell CL, Hoff MN, Balu N, Nguyen XV, Oztek MA, Haynor DR. Making Magnets More Attractive: Physics and Engineering Contributions to Patient Comfort in MRI. Top Magn Reson Imaging. 2020;29: 167–174. doi: 10.1097/RMR.0000000000000246 32541257

[12] Oztek MA, Brunnquell CL, Hoff MN, Boulter DJ, Mossa-Basha M, Beauchamp LH, et al. Practical Considerations for Radiologists in Implementing a Patient-friendly MRI Experience. Top Magn Reson Imaging. 2020;29: 181–186. doi: 10.1097/RMR.0000000000000247 32511199

[13] Makary MS, da Silva A, Kingsbury J, Bozer J, Dowell JD, Nguyen XV. Noninvasive Approaches for Anxiety Reduction During Interventional Radiology Procedures. Top Magn Reson Imaging. 2020;29: 197–201. doi: 10.1097/RMR.0000000000000238 32472820

[14] Ajam AA, Tahir S, Makary MS, Longworth S, Lang EV, Krishna NG, et al. Communication and Team Interactions to Improve Patient Experiences, Quality of Care, and Throughput in MRI. Top Magn Reson Imaging. 2020;29: 131–134. doi: 10.1097/RMR.0000000000000242 32568975

[15] Salazar G, Quencer K, Aran S, Abujudeh H. Patient satisfaction in radiology: qualitative analysis of written complaints generated over a 10-year period in an academic medical center. J Am Coll Radiol. 2013;10: 513–517. doi: 10.1016/j.jacr.2013.03.013 23827003

[16] Kapoor N, Yan Z, Wang A, Wickner P, Kachalia A, Boland G, et al. Improving Patient Experience in Radiology: Impact of a Multifaceted Intervention on National Ranking. Radiology. 2019;291: 102–109. doi: 10.1148/radiol.2019182307 30667330

[17] Pahade JK, Trout AT, Zhang B, Bhambhvani P, Muse VV, Delaney LR, et al. What Patients Want to Know about Imaging Examinations: A Multiinstitutional U.S. Survey in Adult and Pediatric Teaching Hospitals on Patient Preferences for Receiving Information before Radiologic Examinations. Radiology. 2018;287: 554–562. doi: 10.1148/radiol.2017170592 29436946

[18] Boos J, Fang J, Snell A, Hallett D, Siewert B, Eisenberg RL, et al. Electronic Kiosks for Patient Satisfaction Survey in Radiology. AJR Am J Roentgenol. 2017;208: 577–584. doi: 10.2214/AJR.16.16974 28004975

[19] Nairz K, Bohm I, Barbieri S, Fiechter D, Hosek N, Heverhagen J. Enhancing patient value efficiently: Medical history interviews create patient satisfaction and contribute to an improved quality of radiologic examinations. PLoS One. 2018;13: e0203807. doi: 10.1371/journal.pone.0203807 30256840 PMC6157877

[20] European Society of Radiology (ESR). Patient survey of value in relation to radiology: results from a survey of the European Society of Radiology (ESR) value-based radiology subcommittee. Insights Imaging. 2021;12: 6-020–00943-x. 33411144 10.1186/s13244-020-00943-x PMC7790952

[21] Ajam AA, Xing B, Siddiqui A, Yu JS, Nguyen XV. Patient Satisfaction in Outpatient Radiology: Effects of Modality and Patient Demographic Characteristics. J Patient Exp. 2021;8: 23743735211049681. doi: 10.1177/23743735211049681 34660888 PMC8516377

[22] Bartlett JW, Morris TP. Multiple imputation of covariates by substantive-model compatible fully conditional specification. Stata Journal. 2015;15: 437–456.

[23] Martinez JR, Nakonezny PA, Batty M, Wells J. The Dimension of the Press Ganey Survey Most Important in Evaluating Patient Satisfaction in the Academic Outpatient Orthopedic Surgery Setting. Orthopedics. 2019;42: 198–204. doi: 10.3928/01477447-20190625-03 31323103

[24] Blank AT, Shaw S, Wakefield CJ, Zhang Y, Liu WJ, Jones KB, et al. What factors influence patient experience in orthopedic oncology office visits? World J Clin Oncol. 2020;11: 136–142. doi: 10.5306/wjco.v11.i3.136 32257844 PMC7103527

[25] Compton J, Glass N, Fowler T. Evidence of Selection Bias and Non-Response Bias in Patient Satisfaction Surveys. Iowa Orthop J. 2019;39: 195–201. 31413694 PMC6604521

